# Cancer-associated inflammation: pathophysiology and clinical significance

**DOI:** 10.1007/s00432-022-04399-y

**Published:** 2022-10-19

**Authors:** Piotr Pęczek, Monika Gajda, Kacper Rutkowski, Marta Fudalej, Andrzej Deptała, Anna M. Badowska-Kozakiewicz

**Affiliations:** 1 Department of Cancer Prevention, Students’ Scientific Organization of Cancer Cell Biology, Medical University of Warsaw, Warsaw, Poland; 2 Department of Cancer Prevention, Medical University of Warsaw, Erazma Ciołka 27, Warsaw, Poland; 3 Department of Oncology and Haematology, Central Clinical Hospital of the Ministry of Interior and Administration, Warsaw, Poland

**Keywords:** Oncology, Cancer, Inflammation, Immunotherapy

## Abstract

**Purpose:**

Cancer cells, despite stemming from the own cells of their host, usually elicit an immune response. This response usually enables elimination of cancer at its earliest stages. However, some tumors develop mechanisms of escaping immune destruction and even profiting from tumor-derived inflammation.

**Methods:**

We summarized the roles of different immune cell populations in various processes associated with cancer progression and possible methods of reshaping tumor-associated inflammation to increase the efficacy of cancer therapy.

**Results:**

Changes in various signaling pathways result in attraction of immunosuppressive, pro-tumorigenic cells, such as myeloid-derived suppressor cells, tumor-associated macrophages, and neutrophils, while at the same time suppressing the activity of lymphocytes, which have the potential of destroying cancer cells. These changes promote tumor progression by increasing angiogenesis and growth, accelerating metastasis, and impairing drug delivery to the tumor site.

**Conclusion:**

Due to its multi-faceted role in cancer, tumor-associated inflammation can serve as a valuable therapy target. By increasing it, whether through decreasing overall immunosuppression with immune checkpoint inhibitor therapy or through more specific methods, such as cancer vaccines, oncolytic viruses, or chimeric antigen receptor T cells, cancer-derived immunosuppression can be overcome, resulting in immune system destroying cancer cells. Even changes occurring in the microbiota can influence the shape of antitumor response, which could provide new attractive diagnostic or therapeutic methods. Interestingly, also decreasing the distorted tumor-associated inflammation with non-steroidal anti-inflammatory drugs can lead to positive outcomes.

## Introduction

A decade ago, tumor-promoting inflammation and avoiding immune destruction were added to the list of hallmarks of cancer, the group of molecular properties that characterize malignant tumors. It highlights the importance of tumor-associated inflammation and immunosuppression in understanding cancer biology, development of new methods of treatment, and optimization of the existing ones (Hanahan and Weinberg 2011).

In normal circumstances, malignant cells should be detected and destroyed by the immune system as soon as they appear, in a process called immune surveillance. However, this defense mechanism sometimes fails and cells evading immunity are selected, which is called immunoediting, and give rise to detectable cancers. Cancer cells not only evade detection, but also exert an immunosuppressive effect, which remodels the tumor microenvironment (TME), which begins to promote cancer growth instead of inhibiting it. Although inflammation is still present at the tumor site, it no longer serves its purpose (Ostrand-Rosenberg 2008). Both innate immune cells [macrophages, neutrophils, dendritic cells (DCs), innate lymphoid cells (ILCs), myeloid-derived suppressor cells, and natural killer (NK) cells] and adaptive immune cells (T cells and B cells) present at the tumor site modulate tumor progression, invasion, and metastasis through secreted cytokines and other mechanisms (Hinshaw and Shevde 2019). Moreover, multiple oncological therapies strengthen these unfavorable changes in the TME, which can be responsible for the decrease of therapy effectiveness over time (Shaked 2019). The dual, pro-, and anti-tumor nature of immune response to cancer makes it a valuable target for therapy and understanding it can also help improve the effectiveness of more traditional cancer treatments (Molinaro et al. 2018).

In this review, we recapitulate the role of the most influential factors and cells shaping the immune microenvironment of cancer, the processes influenced by cancer-associated inflammation, and the methods of targeting and reshaping it to restore anti-tumor immune response and improve the effectiveness of other treatments. However, this is just a brief overview, meant to provide general understanding of this complex matter, and the precise mechanisms of the relationship between the immune system and cancer are not described in detail.

## Elements of tumor-associated inflammation

### The role of genetic aberrations in cancer cells

Genetic abnormalities are required for a tumor to develop. Besides their role in dysregulation of cellular processes like cell cycle, apoptosis, migration, and survival, they also correlate with tumor immune landscape, which has been extensively studied in recent years (Wellenstein and Visser 2018; Rooney et al. 2015). Tumor cells’ intrinsic genetic events lead to the activation of certain transcription factors, e.g., nuclear factor-κB (NF-κB), signal transducer and activator of transcription 3 (STAT3), and hypoxia-inducible factor 1α (HIF1α). As a result, various cytokines, chemokines, growth factors, prostaglandins, reactive oxygen, and nitrogen species are secreted from the transformed cells. These mediators contribute to the recruitment of leukocytes, which also produce a plethora of inflammatory mediators, triggering additional inflammatory signals in other tumor, stromal, and immune cells. The amplified cancer-related inflammatory cascade facilitates tumor proliferation, angiogenesis, invasion, metastasis, and immune evasion of the malignant cells (Yang and Lin 2017; Mantovani et al. 2008; Cunha et al. 2019; Nakamura and Smyth 2017).

For example, *NF1* loss in glioma cells results in increased presence of macrophages (Wang et al. 2017a). Another study showed that in ER-negative, basal-like breast tumors loss of heterozygosity or mutation of *TP53* is associated with lower lymphocytic infiltration compared to those with wild type p53 (Quigley et al. 2015). Mutations in cancer cells sometimes result in expression of neoantigens, followed by presence of neoantigen specific T cells, as observed in melanoma patients (Linnemann et al. 2015). Genetic aberrations can also influence NK cells response, like in tumor neuroblastoma cell lines, where *MYCN* overexpression correlates with lower expression of ligands for the natural killer group 2, member D (NKG2D), and DNAX accessory molecule-1 (DNAM-1) NK-cell-activating receptors (Brandetti et al. 2017). Additionally, an impact on the composition of tumor microenvironment has been observed in thyroid tumors with mutated *BRAF* or *RAS* (Charoentong et al. 2017), as well as using data from The Cancer Genome Atlas, in various tumors with mutations in *PIK3CA*, *MET*, *VHL*, or *STK11* (Wellenstein and Visser 2018; Rooney et al. 2015). Numerous other studies report that alterations in receptor tyrosine kinases (RTKs), such as rearranged during transfection (RET) activation and increased epidermal growth factor receptor (EGFR) signaling, as well as oncogenic rat sarcoma virus protein (Ras) contribute to pro-inflammatory cytokine expression (Yang and Lin 2017). Similar results have been observed in tumors with mutated p53, APC, and transforming growth factor beta (TGFβ), establishing their role in shaping tumor immune milieu (Yang and Lin 2017; Agupitan et al. 2020). These are just a few examples of the impact that genetic aberrations have on the immune contexture of tumors. From the therapeutic point of view, it is important to assess whether a direct link between cancer genetic makeup and its immune landscape exists, since it may enable development of new personalized strategies for patients (Wellenstein and Visser 2018).

### MDSCs

Myeloid derived suppressor cells (MDSCs) are bone marrow-derived, highly heterogeneous, immature cells that largely contribute to the immunosuppression within the TME, compromising both innate and adaptive immune responses (Hinshaw and Shevde 2019; Emami Nejad et al. 2021; Vito et al. 2020; LaGory and Giaccia 2016; Ostrand-Rosenberg and Fenselau 2018). MDSCs can be divided into groups: monocytic MDSCs (M-MDSCs), polymorphonuclear MDSCs (PMN-MDSCs), and early stage MDSCs (eMDSCs). M-MDSCs and PMN-MDSCs present at the tumor site show more anti-inflammatory properties than MDSCs outside of the TME. MDSCs facilitate metastasis by enhancing angiogenesis and initiating development of the pre-metastatic niche (Hinshaw and Shevde 2019). They increase macrophage polarization towards M2 phenotype, attract regulatory T lymphocytes (Tregs), decrease cytotoxicity of NK cells, and suppress T-cell function, favoring tumor progression. For example, MDSCs contribute to breast cancer growth and metastasis through inducing T-cell exhaustion (Zhu et al. 2017a). A variety of overlapping pathways drive to the MDSCs infiltration of the tumor site (Ostrand-Rosenberg and Fenselau 2018). As an example, C-X-C motif chemokine ligand 5 (CXCL5) is a tumor-secreted chemokine that draws MDSCs expressing C-X-C motif chemokine receptor 2 (CXCR2) and, in consequence, its suppression disrupts tumor development (Wang et al. 2016). In hepatocellular carcinoma, these myeloid-derived cells can be accumulated in the hypoxic TME through C–C motif chemokine ligand 26 (CCL26) (Chiu et al. 2016) or through ectonucleoside triphosphate diphosphohydrolase 2 (ENTPD2) (Chiu et al. 2017). In renal cell carcinoma, the presence of PMN-MDSCs is correlated with the secretion of interleukin-1β (IL-1β), IL-8, CXCL-5, and macrophage inflammatory protein-1α (Mip-1α) (Najjar et al. 2017). It is noteworthy that MDSCs reduce effectiveness of numerous therapeutic strategies, such as immune checkpoint inhibition therapy, and hence, a number of current studies are focusing on eliminating them from the TME (Ostrand-Rosenberg and Fenselau 2018).

### Tumor-associated macrophages

**Fig. 1:**
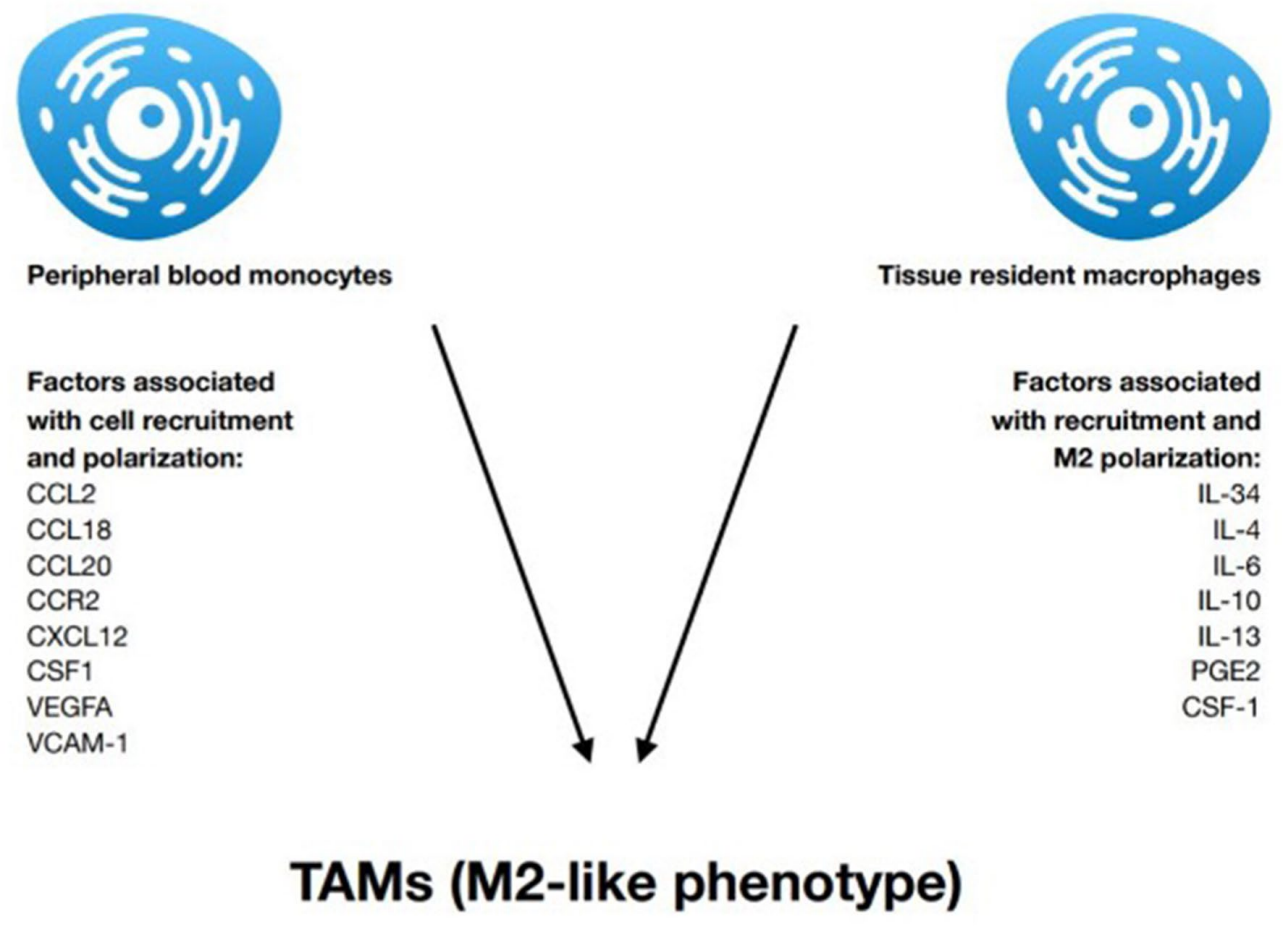
Summary of the most important cytokines associated with the recruitment of peripheral and resident macrophages to the tumor site and their polarization towards M2-like phenotype

Macrophages are immune cells present in various tissues, where they seek for signs of pathogens or damage. If found, they stimulate lymphocytes and other immune cells to respond (Murray and Wynn 2011). They can be divided into two populations; either M1 which are induced by type 1T helper (Th1) cytokines or by bacterial lipopolysaccharide recognition and display pro-inflammatory activity, or M2, also called alternatively activated macrophages, induced by type 2T helper (Th2) cytokines, representing anti-inflammatory, pro-angiogenic, and pro-fibrotic properties (Shapouri-Moghaddam et al. 2018). They are one of the most abundant cell types in TME and are estimated to account for up to 50% of cancer tissue mass (Zhang et al. 2019). Macrophages present in tumor, under suitable conditions, are remodeled into tumor-associated macrophages (TAMs). IL-4 and IL-13 cytokines are recognized as strong inducers of an alternative (M2) macrophage activation. Additionally, IL-34 overexpression by osteosarcoma, through recruiting M2-like macrophages, is associated with increased tumor growth, vascularization, and metastasis (Szebeni et al. 2017). TAMs present similar activity and characterization to M2 macrophages; nevertheless, they also share M1 signature polarization (Chavez-Galan et al. 2015). Signals in the tumor milieu [IL -4, IL -6, IL -10, prostaglandin E2 (PGE2), colony-stimulating factor 1 (CSF-1), and TGF-β] polarize macrophages into alternative, pro-inflammatory, pro-angiogenic, and immunosuppressive, protumoral M2-like cells (Caronni et al. 2015). Superior lactate level in the TME also drives macrophages differentiation towards M2 phenotype via extracellular signal-regulated kinase/signal transducer and activator of transcription 3 (ERK/STAT3) signaling activation. The inhibition of ERK/STAT3 can cause an abatement in breast cancer cell proliferation and migration induced by lactate-activated macrophages (Mu et al. 2018). In hepatocellular carcinoma (HCC), TAMs facilitate the expansion of cancer cells by secreting IL-6 activating STAT3. Moreover, TAMs induce epithelial mesenchymal transition and promote cellular migration through the janus kinase 2/signal transducer and activator of transcription 3 (JAK2/STAT3) signaling pathway. These findings suggest that STAT3 could be considered a target molecule (Chavez-Galan et al. 2015). TAMs impose pro-angiogenic, pro-invasive, and immunosuppressive effect on the tumor and can, therefore, be viewed as a promising foothold in tumor immunotherapy (Zhou et al. 2020a). TAMs arise from two different cell populations. Presumably, tissue-resident macrophages are first to be adjusted by the tumor cells to embody pro-tumor M2-like phenotype. Supplementary to that, peripheral blood monocytes are being recruited to the TME and polarized into M2-like TAMs (Zhou et al. 2020b). This occurs as a response to chemokines and growth factors produced by stromal and tumor cells in the TME. These include CCL2, CSF1, vascular endothelial growth factor A (VEGF-A), CCL18, CCL20, and CXCL12 (Yang and Zhang 2017). Also, vascular cell adhesion molecule-1 (VCAM-1) overexpression is associated with higher macrophages’ infiltration (Zhang et al. 2019). More specifically, TAMs originate from circulating Ly6C^+^ CCR2^+^ monocytes or from tissue-resident macrophages that originate from CXC3CR1^+^ Kit^+^ erythromyeloid progenitors from yolk sac or murine fetal liver (murine model) independently of bone marrow (Wu and Zhang 2020). CCL2 was initially discovered as a tumor-derived chemoattractant which recruits monocytes/macrophages to tumor tissue (Szebeni et al. 2017). Targeting CCR2–CCL2 axis, which is the major chemokine axis responsible for monocyte recruitment, is one of the strategies related to TAMs currently under clinical trial (Wu and Zhang 2020). Anti-CCL2 antibody (Carlumab) was shown to inhibit growth of glioma, colon, prostate, and melanoma cancers in animal models. However, further clinical trials indicated that Carlumab did not block the CCL-2/C–C chemokine receptor 2 (CCR-2) axis, nor did it show anti-tumor activity in metastatic castration-resistant prostate cancer. Consequently, further clinical investigation of whether Carlumab’s success in animal model can also be achieved with human patients is necessary. Successful inhibition of CCL-2 can be achieved by blocking IL-6. Anti-IL-6 antibody (Siltuximab) is proven to have an anti-cancer effect and decreases not only CCL-2, but also VEGF and CXCL-12 expression (Sawa-Wejksza and Kandefer-Szerszen 2018) (see Fig. 1).

### Neutrophils

Similarly to macrophages, tumor-associated neutrophils (TANs) can also be divided into immunostimulatory N1 neutrophils and immunosuppressive N2 neutrophils. One of the mechanisms of neutrophil attraction to the TME is the secretion of CXCL1, CXCL2, CXCL5, and CXCL8 by the malignant cells and the tumor stroma. Of note, the high infiltration of these inflammatory cells correlates with unfavorable prognosis for patients with many different types of cancer. Neutrophils enhance tumor growth, angiogenesis, and metastatic potential. For example, they produce CCL17 to recruit Tregs that silence immune response (Liang and Ferrara 2016). TGF-β has been shown to aid the polarization of the neutrophils towards N2 phenotype, characterized by tumor-promoting properties (Emami Nejad et al. 2021; LaGory and Giaccia 2016; Liang and Ferrara 2016). They secrete cytokines, chemokines, reactive oxygen species, reactive nitrogen species, nitric oxide, and matrix metalloproteinases to promote angiogenesis, metastasis, and cancer invasion (Hinshaw and Shevde 2019; Cunha et al. 2019). Interestingly, malignant cells accentuate ejection of the nuclear or mitochondrial DNA by the neutrophils, known as neutrophil extracellular traps (NETs) formation, potentially contributing to metastatic cancer progression (Tohme et al. 2016; Park et al. 2016).

### Lymphocytes

Tumor-infiltrating lymphocytes (TILs) are undoubtedly one of the most important elements of the tumors contexture. Cytotoxic T cells (CTLs) are capable of recognizing tumor-associated antigens and eliminating neoplastic cells by either secreted molecules (such as perforins and granzymes) or death ligands (Martínez-Lostao et al. 2015). High infiltration of lymphocytes is correlated with positive outcome in different cancer entities, such as metastatic melanoma, ovarian, colorectal, and breast cancer (Ferrari et al. 2019). Tumors produce a wide variety of cytokines and chemokines that induce immune cell migration to the TME, but they can also downregulate some signaling pathways to reduce the chemoattraction of inflammatory cells, such as CTLs. There are also multiple tools that cancer cells use to protect themselves from the execution (Woude et al. 2017). Expression of programmed cell death ligand 1 (PD-L1) and cytotoxic T-lymphocyte-associated protein 4 (CTLA-4) that induce exhaustion of T cells appears to be the most remarkable tactic (Woude et al. 2017; Tu et al. 2020). Therapeutic approach targeting these proteins, known as immune checkpoint inhibitor (ICI) therapy, is discussed in another section of this paper. Other strategy of immune evasion depends upon the secretion of indoleamine 2,3-dioxygenase (IDO), which causes tryptophan deficiency and kynureine production, resulting in T-cell apoptosis. Furthermore, previously mentioned in the context of N2 neutrophils, TGF-β produced by cancer cells and other components of the TME inhibit T-cell proliferation and activation, silencing their anti-tumoral activity (Woude et al. 2017). Hypoxia at the tumor site has also been reported to mute anti-tumoral activity of TILs (Emami Nejad et al. 2021; Woude et al. 2017). All in all, malignant cells have the potential to effectively impair CTLs properties, and many strategies interrupting this unfavorable crosstalk are used in clinical trials (Woude et al. 2017).

In contrary to CTLs, Tregs constitute an immunosuppressive subset of T cells, associated with poor outcome in cancer patients, with an exception for colorectal cancer. They are characterized by forkhead box P3 (Foxp3) and cluster of differentiation 25 (CD25) expression and play a crucial role in autoimmunity prevention (Moreno Ayala et al. 2019; Ohue and Nishikawa 2019). In the context of tumor, Tregs inhibit inflammatory response through multiple pathways. They block costimulatory signals from DCs, eliminate CTLs through ICI, and kill antigen‐presenting cells using secreted molecules and death ligands. Moreover, they express immunosuppressive cytokines, consume IL-2, and modulate metabolic interactions at the tumor site by lowering adenosine triphosphate (ATP) levels and inducing IDO expression in the DCs (Ohue and Nishikawa 2019). These highly tumor-promoting properties make Tregs a major challenge in cancer immunotherapy. Current studies are focusing on selectively targeting Tregs to elicit augmented tumor immunity without severe autoimmune reactions (Moreno Ayala et al. 2019; Tanaka and Sakaguchi 2019).

### Hypoxia, lactate, and acidosis

**Fig. 2:**
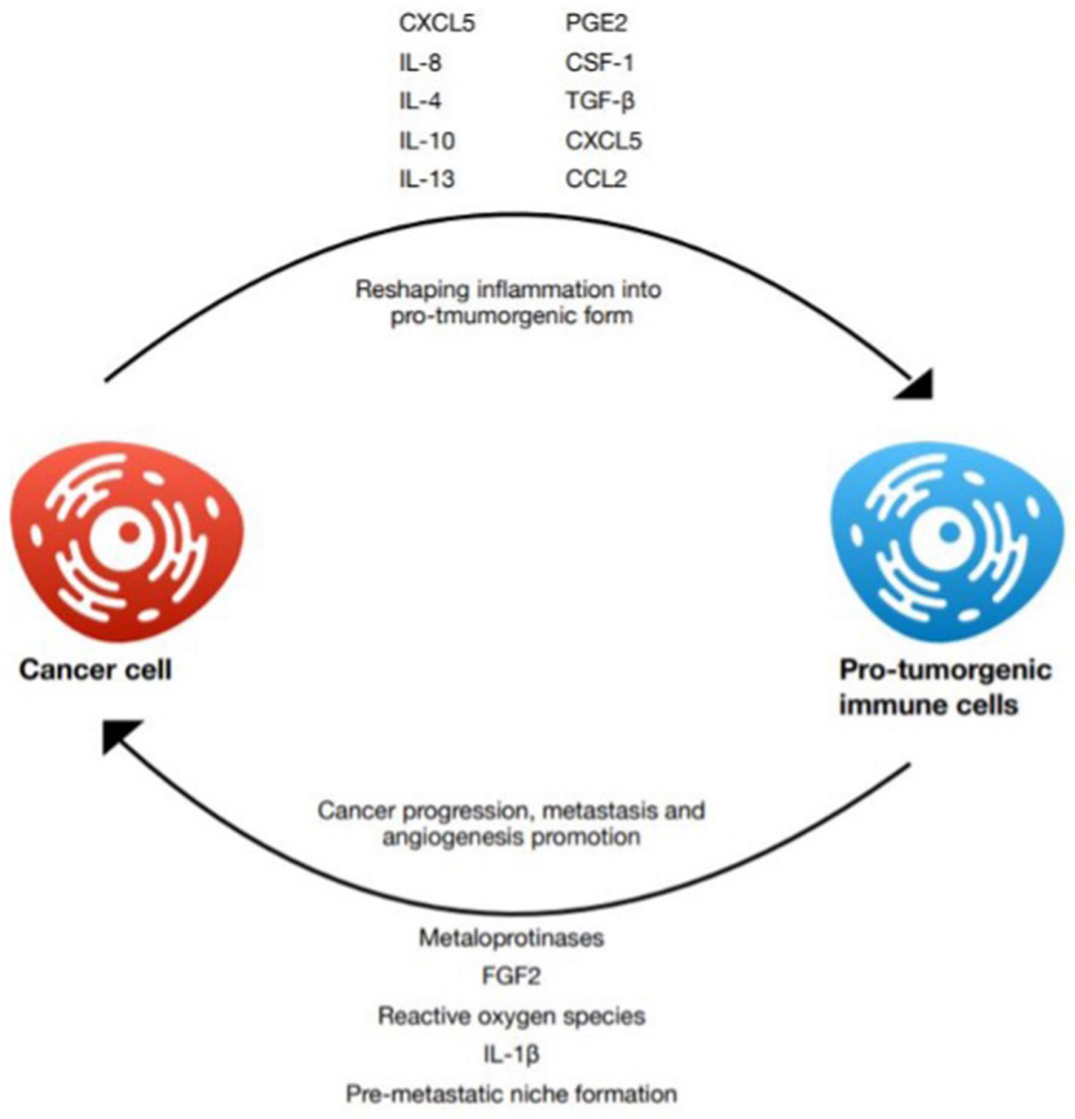
Summary of some of the factors shaping the pro-tumorigenic cancer microenvironment and its influence on cancer progression, metastasis, and angiogenesis

Hypoxia in known to be an important feature of solid tumors, which induces the production of hypoxia-related factors (HIFs), such as VEGF (Goel et al. 2011). Besides its well-established role in inducing formation of a new vasculature, invasiveness, metabolic reprogramming, and therapy resistance, hypoxic environment promotes immunosuppression and facilitates immune evasion (Nakamura and Smyth 2017; Vito et al. 2020; LaGory and Giaccia 2016; Codony and Tavassoli 2021). As a response to low oxygen availability, HIFs become transcriptionally active and stimulate the expression of numerous genes, contributing to various immunomodulating pathways (Vito et al. 2020). The hypoxia enhances glycolysis and glutaminolysis, resulting in high levels of lactate and low pH (from 6.3 to 6.9) at the tumor site (Multhoff and Vaupel 2020; Pérez-Tomás and Pérez-Guillén 2020). Acidified TME increases the tumor proliferation, survival, neovascularization, metastasis, as well as immunosuppression by altering infiltrating immune cells (Pérez-Tomás and Pérez-Guillén 2020). High extracellular level of lactic acid dampens the immune response by reducing the number of M1 macrophages, decreasing the effectiveness of cytotoxic T and NK cells, and suppressing the release of pro-inflammatory cytokines (Colegio et al. 2014). Function of M2 macrophages and Treg cells seems to be supported, while lowered proliferation of T cells and their apoptosis is observed (Multhoff and Vaupel 2020). Hypoxia increases the production of lactic acid, resulting in TAMs polarization towards M2 phenotype in an HIF-1α dependent mechanism (Colegio et al. 2014). Tumor cells under the hypoxic conditions recruit MDSCs and promote their differentiation and cancer-promoting function (Emami Nejad et al. 2021; Vito et al. 2020). Hypoxia also aids neutrophil survival, favoring tumor progression and stimulating angiogenesis (Liang and Ferrara 2016; Multhoff and Vaupel 2020). Furthermore, it modulates immune checkpoints by enhancing the transcription of PD-L1 in hypoxic malignant cells, macrophages, and MDSCs, as well as inducing the expression of programmed cell death 1 (PD-1), lymphocyte activating gene 3 (LAG3) and CTLA-4 (Emami Nejad et al. 2021). The relevance of hypoxic conditions in cancer progression has been used to develop new therapeutic strategies, such as HIF-inhibitors, hypoxia-activated prodrugs, and anti-angiogenic agents. Despite all the clinical advances, only a few of them has proven effective in clinical practice (Codony and Tavassoli 2021). Of note, since lactate appears to be a crucial signaling molecule in the TME, further studies are likely to offer a new approach for cancer treatment (Pérez-Tomás and Pérez-Guillén 2020) (see Fig. 2).

## Processes influenced by inflammation

### Angiogenesis

Immune cells are greatly involved in the formation of new vessels at the tumor site. Metalloproteinases (MMPs) and cathepsins produced by TAMs degrade basement membrane and matrix tissue. Additionally, pro-angiogenic factors, such as VEGF, platelet-derived growth factor (PDGF), fibroblast grow factor 2 (FGF2), and chemokines CCL2 and CXCL8, stimulate endothelial cell proliferation and migration, thus providing the vascular network necessary for tumor growth and cancer cells dissemination (Petty and Yang 2017). Shortage in oxygen delivery is a common feature of solid tumors. Hypoxic TAMs promote tumor progression and growth of cancer cells via upregulation of grow factors, such as FGF2, PDGF, and VEGF (Henze and Mazzone 2016). Wnt family member 7b (Wnt7b) synthetized by TAMs targets vascular endothelial cells and stimulates expression of VEGF. Pro-matrix metalloproteinase-9 (proMMP-9) was also shown to play a crucial role in angiogenesis and metastasis (Guo et al. 2016). Targeting MMP9 by a bisphosphonate, zoledronic acid, was proven to inhibit the angiogenesis in cervical carcinoma model (Wu and Zhang 2020). VEGF-A implies new, abnormal vessels’ formation comprising excessive branching, dead-end vessels, and increased vessel leakiness. All together, these anomalies in vessel formation result in the impairment of hemodynamic and drug delivery (Ruffell and Coussens 2015). It has been proven that macrophage infiltration is higher in malignant tumors. An interrelationship was found between macrophage infiltration and microvessels’ density. In malignant tumors, hypervascularity was simultaneous with high macrophages’ infiltration (Badawi et al. 2015). VEGF antagonists implicate vessel normalization; they have also been shown to enhance chemotherapeutics uptake (Ruffell and Coussens 2015). STAT3 activation upregulates the production of pro-angiogenic factors, such as VEGF. Cell–cell interaction between cancer cells and macrophages is crucial for STAT3 activation. NF-kB was shown to also be involved in angiogenesis induced by these cell–cell interactions (Komohara et al. 2016). Inhibiting VEGF pathway with monoclonal antibodies (for example bevacizumab) is one of the techniques that help controlling tumor growth and angiogenesis (Salmaninejad et al. 2019). Anti-VEGF monotherapy alone presented lack of meaningful survival benefits. However, large, randomized phase 3 clinical trials of bevacizumab have proven significant improvement in overall survival in many cancers when combined with systemic chemotherapy contrarily to systemic chemotherapy alone (Goel et al. 2011). In the glioblastoma murine model, it was shown that combined inhibition of VEGF receptors and angiopoietin-2 significantly improves survival over inhibition of VEGF receptor alone (Peterson et al. 2016). Disruption in oxygen delivery, which causes hypoxia, is a common feature of tumors above certain size. Hypoxic regions are rich in factors, such as CCL2, CCL5, CSF1, VEGF, and others. Those factors attract macrophages to hypoxic tumor compartments. Consequently, the mobility of macrophages is decreased due to a diminished CCR2, CCR5, and Neutropilin-1 (NRP1) expression which disrupts signaling pathways (Henze and Mazzone 2016). Hypoxia is one of the circumstances that promote angiogenesis by inhibiting ubiquitination of HIFs. HIF-1 is a heterodimer transcription factor promoting angiogenesis by activating expression of pro-angiogenic mediators (Fu et al. 2020a). In normoxemia surrounding HIF-1α undergoes ubiquitylation leading to proteasomal degradation; under hypoxic conditions, ubiquitylation is impaired (Fu et al. 2020a). This results in transcription of HIF-driven hypoxia-related genes such as VEGF (Goel et al. 2011). NRP1 plays a crucial role in entering TAMs into hypoxic niches; meanwhile, loss of NRP1 restores anti-tumor immunity and inhibits angiogenesis (Fu et al. 2020a). TAMs add up to vasculogenesis by releasing IL-6 which activates JAK/STAT3 pathways in endothelial progenitor cells (Zhu et al. 2017b).

### Immunosuppression

**Fig. 3:**
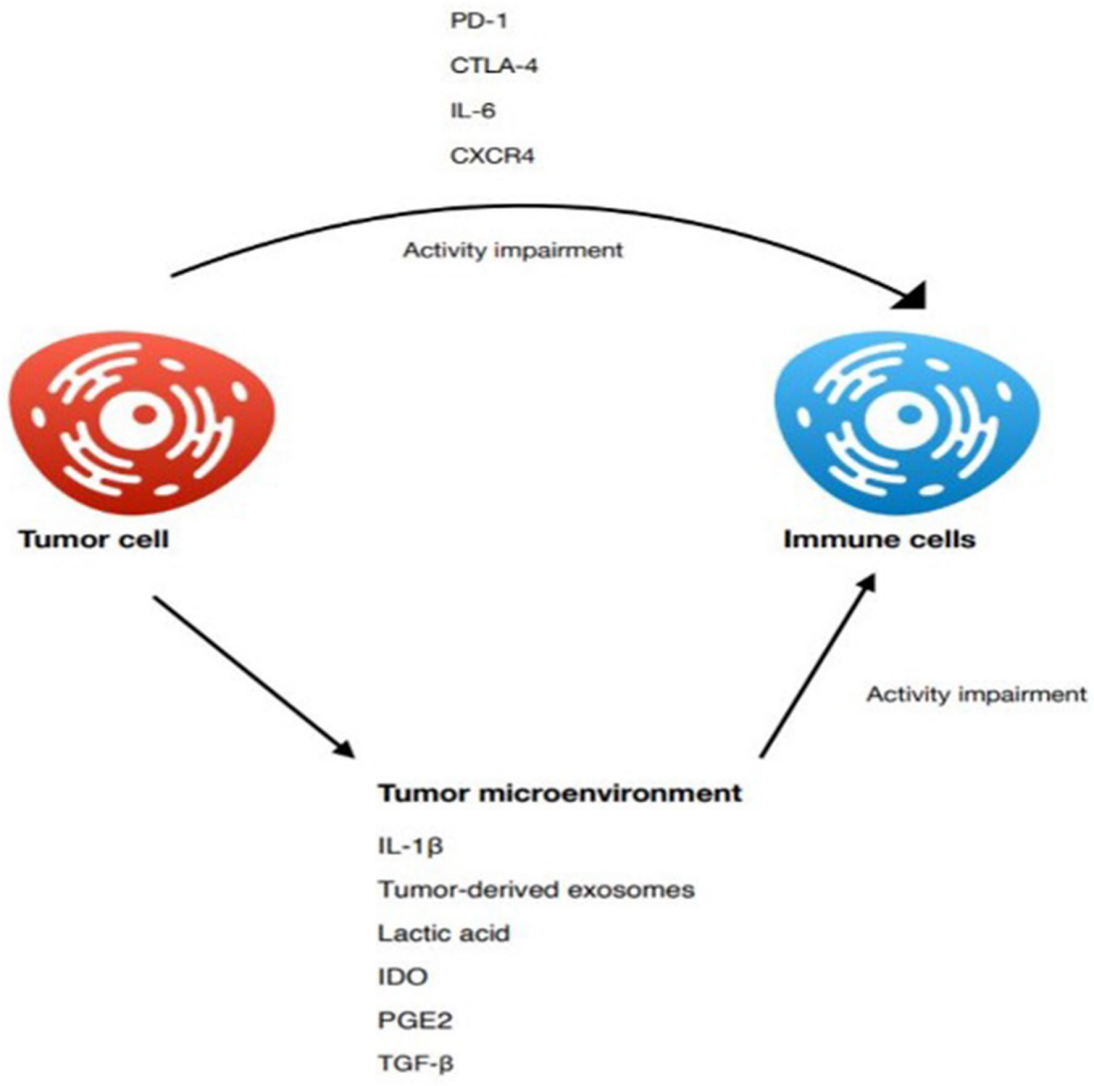
Summary of the factors promoting tumor-associated inflammation derived from the tumor microenvironment or directly from cancer cells

Escaping from immune surveillance is an essential step for the tumor to grow. A way to create an immunosuppressive habitat is through ensuring a high concentration of immunosuppressive cells (Tregs, MDSCs), which correlates with poor prognosis and is a major obstacle for immunotherapy (Shimizu et al. 2018). Inhibiting CXCR4 by Plerixafor (AMD3100) can lead to a decrease in intratumoral regulatory T cells infiltration. When combined with anti-PD-1 antibody, the therapy significantly inhibited tumor growth and prolonged the survival of tumor bearing mice. The anti-tumor effect was also present in monotherapy with AMD3100 or anti-PD1, but the results were less impressive (Zeng et al. 2019). Immunosuppressive habitat in TME inhibits the NK cells from exerting an anti-tumor response. The tumor and tumor-associated cells secrete several factors that impair NK cells activation, such as IL-6, TGF-B, PGE2, and IDO (Terren et al. 2019). Interleukin-1β (IL-1β) present in the TME recruits myeloid cells, limits anti-tumor effects of chemotherapy, and promotes tumor invasiveness and immunosuppression. Treatment with anti-IL-1β antibodies followed by anti-PD-1 antibodies completely abrogated tumor progression in the model of orthotopically introduced 4T1 breast cancer (Kaplanov et al. 2019). Further factors contributing to the immunosuppressive habitat of tumor tissue are tumor-derived exosomes (TEX). They can stimulate angiogenesis, inhibit immune cell proliferation, induce apoptosis of activated CD8^+^ T lymphocytes, suppress NK cell activity, promote regulatory T cells, or interfere with monocyte differentiation along with promoting MDSC expansion. Exosomes released by stoma cells have the ability to stimulate metastasis formation as well as promote tumor cell proliferation and apoptosis inhibition, not to mention TEX competence to interfere with immunotherapies. TEX were proven to interfere with therapeutic activity of Trastuzumab, which is frequently used in breast cancer therapy (Olejarz et al. 2020; Whiteside 2017). Impaired oxygen delivery to tumor leads to elevated lactic acid level in the TME. Lactic acid is one of the many factors responsible for suppressing anti-cancer immunity. In elevated levels, it spoils monocyte differentiation into dendritic cells and decreases their antigen-presentation functions. Second, it inhibits anti-tumor activities of immune effector cells including natural killer cells and cytotoxic T cells. Furthermore, it promotes the infiltration of immunosuppressive cells such as M2 TAMs, N2 neutrophils, MDSC, and regulatory T cells, all of which contribute to cancer immune escape (Wang et al. 2020) (see Fig. 3).

### Metastases

Metastases are the major cause of mortality in cancer patients, and hence, understanding each step of tumor metastasis is crucial for developing new therapeutic strategies. Hypothesis of “seed and soil” not only is auxiliary for understanding the tumor metastasis but also provides an explanation for organotropism (Liu and Cao 2016). The concept of the primary tumor forming a “fertile soil” for circulating tumor cells (CTCs) (“seeds”) has attracted researchers’ attention. TAMs can easily remodel extracellular matrix (ECM) by secreting extracellular proteases. Remodeling of ECM has been recognized as essential for cancer cell invasion, migration, and metastasis. Additionally, interactions between TAMs and tumor cells can lead to formation of a protrusions known as invadopodia in cancer cells, which are able to degrade ECM (Sanchez et al. 2019). Depletion of macrophages decreases the metastatic potential of disseminated cancer cells (Doak et al. 2018). Epithelial-to-mesenchymal transition (EMT) is crucial for tumor cells invasiveness and metastasis. TAMs secrete many cytokines involved in EMT induction, such as TGF-B and IL-6 (Guo et al. 2016). EMT plays a critical role in metastasis formation; however, it is inconsistent with the fact that metastatic tumors share the epithelial heritage of primary tumors. Several studies have shown that opposite phenomenon known as mesenchymal-to-epithelial transition (MET) might be crucial when it comes to forming a metastatic lesion. Inhibiting MET along with EMT could be another promising approach for cancer therapy (Liu et al. 2017). Lysyl oxidase, placental growth factor (PIGF), and exosomes produced by primary tumors prepare a pre-metastatic niche for disseminated tumor cells. These factors released by the tumor induce the recruitment of CD11b + VEGFR1 + myeloid cells assist metastases formation (Nielsen and Schmid 2017). Another mechanism by which primary tumor can inaugurate formation of pre-metastatic niche is via activating resident macrophages through soluble and exosomal signaling. In the liver, Kupffer cells mop up TGF-exosomes produced by the primary tumor, which then activates secretion of fibronectin, helping in recruitment of bone marrow-derived macrophages (BMDM). BMDM excrete granulin which stimulate fibroblast secretion of periostin. This generates a fibrotic environment which supports colonization of the hepatic tissue by tumor cells. Interaction between tumor cell and metastasis-associated macrophages further promotes metastasis formation by secretion of grow factors, and encourages angiogenesis and immunosuppressive environment formation (Doak et al. 2018). Moreover, it was shown that primary tumor cells (of colorectal carcinoma) secrete VEGF-A, stimulating TAMs to produce CXCL1, which recruits CXCR2^+^ MDSCs to form a pre-metastatic niche that promotes liver metastasis. This knowledge may offer CXCR2 as a new target to treat and prevent development of colorectal carcinoma metastases (Wang et al. 2017b). Similarly, in non-small cell lung cancer, tumor-derived IL-6 recruits immune checkpoint molecule presenting MDSCs, which exert a systemic effect and promote brain metastases. This suggests that monitoring immunosuppressive factors in peripheral blood can be beneficial during cancer treatment (Li et al. 2019). Cytochrome P450 (CYP) 4A^+^ TAMs were positively associated with metastasis, pre-metastatic niche formation, and poor prognosis in breast cancer patients. Inhibition of CYP4A reduced lung pre-metastatic niche formation (decrease in VEGFR1^+^ myeloid cell recruitment and pro-metastatic protein expression); in addition, TAMs phenotype became distant from M2 phenotype in spontaneous metastasis models of 4T1 breast cancer and B16F10 melanoma (Chen et al. 2017).

### Inflammation and tumor drug delivery

To work properly and kill tumor cells, systematically administered drugs must be distributed through the tumor vascular network, cross vessel walls, penetrate interstitial space, and reach tumor cells at sufficient concentration (Khawar et al. 2015). Tumor vasculature is highly disorganized, with malformed, dilated, and tortuous vessels. Some of tumor blood vessels are not even totally lined with endothelium; instead, they are formed like a mosaic with mural-like cancer cells mimicking the function of normal endothelial cells (Zhao et al. 2020). Abnormal structure and function of tumor stroma, known as desmoplasia, hinders sufficient drug delivery. Dense fiber architecture of the tumor compresses intratumoral blood vessels, reducing the administration of the drug to the tumor. Hyper-permeable, immature blood vessels, which arise due to upregulation of pro-angiogenic factors, are another cause of insufficient drug delivery (Gkretsi et al. 2017). Interstitial fluid pressure (IFP) in the tumor is much higher than in normal, healthy tissue. This is caused by cell proliferation, presence of highly crosslinking collagen, high vascular permeability, lack of pericyte coverage, and other factors such as increased secretion of grow factors. Unfortunately, high IFP also stimulates tumor cell proliferation (Muntimadugu et al. 2017). Leaky tumor vasculature creates an opportunity for macromolecular drug delivery system such as micelles, liposomes, gold, or carbon nanoparticles. Their size is big enough to avoid renal or hepatic clearance but still small enough to extravasate from abnormal and leaky tumor vessels (Khawar et al. 2015). Tumor vessels’ hyper-permeability, compressed blood vessels, and high extracellular matrix density interrupt tumor perfusion resulting in hypoxic and acidic environment which can sustain tumor invasion, metastasis, and grant therapy resistance. Plasma leakage, associated with hyper-permeability of tumor vessels, cannot be drained by dysfunctional lymphatic system, dense ECM halts it from percolating out of the tumor, altogether leading to an elevation of interstitial fluid pressure. IFP together with hypoperfusion are hallmarks of the tumor microenvironment (Stylianopoulos et al. 2018).

## Therapies that remodel the inflammatory and immune response

### Immune checkpoint inhibitors (ICI)

Immune checkpoint molecules, most notably PD-1 and CTLA-4, are important regulators of immune response, meant to prevent autoimmunization (Buchbinder and Desai 2016). However, cancer cells often exploit this mechanism by presenting PD-1 or CTLA-4 molecules on their surface, thereby preventing their elimination by immune cells (Tu et al. 2020). Over the last decade, several monoclonal antibodies targeting these proteins have been devised, making it possible to overcome tumor-induced immunosuppression and promote anti-tumor immune response (Darvin et al. 2018). Despite ICI therapy having better safety profile than chemotherapy, it can result in inflammatory adverse effects spanning various organ systems. It is controversial whether the onset of those side effects increases the response to treatment (Postow et al. 2018). The greatest challenge in ICI therapy is relatively low response rates. In some cases, as many as 50% of patients fail to respond to his therapy (Rotte 2019). This can be caused by mutations in other immune signaling pathways or antigen presenting apparatus, low expression of mutated proteins on cancer cell surface or insufficient immune cell infiltration (Jenkins et al. 2018). It has been shown that a combination of CTLA-4 and PD-1 blockers can increase treatment response and can be a valuable strategy in treatment of advanced or metastatic cancers (Rotte 2019). There are also methods of increasing ICI treatment success rate without increasing the risk of adverse effects, for example by modifying the microbiome composition, discussed further in the next paragraph. Other ways of enhancing ICI efficacy are discussed in the following paragraphs.

### Microbiota

The microbiota is composed of commensal bacteria living on the epithelial barriers of the host, most abundantly in the intestine. Its influence extends to the whole human body, and is related to various physiological functions, such as immune response and metabolism (Thaiss et al. 2016). Cancer-associated inflammation and the microbiota influence each other in different ways, which can correlate with the effectiveness of treatment (Roy and Trinchieri 2017). Changes in the microbiota caused by inflammation could also act as a biomarker for cancer diagnosis and prognosis (Villeger et al. 2018).

The gut microbiota affects various anti-cancer treatments (Roy and Trinchieri 2017). Because of its immunomodulating effect, it can increase the response to ICI therapy, not only in colon cancer, but also in cancers outside of the digestive tract, such as melanoma. It has been found that immunotherapy responders are often characterized by greater microbiome diversity and relative abundance of certain groups, such as *Ruminococcaceae* or *Bifidobacteria* (Gopalakrishnan et al. 2018; Sivan et al. 2015). Assessing the microbiome-derived metabolome has been suggested as a potential predictor of response to the immunotherapy (Malczewski et al. 2020). The gut microbiota also plays a role in chemotherapy. It can influence metabolism and efficacy of chemotherapeutic drugs and also lower their toxicity, even in systemically delivered drugs (Roy and Trinchieri 2017). Different studies suggested that manipulating the composition of the microbiota with probiotics or antibiotics can have beneficial effects on cancer treatment and prognosis (Roy and Trinchieri 2017; Sivan et al. 2015; Shang et al. 2020).

On the other hand, tumor-induced inflammation can also modulate the bacterial composition of the microbiome, both in lungs and the gut, and cause dysbiosis, which can even promote cancer progression. Inflammatory cells release reactive nitrogen species, which generally have an anti-microbial effect, but some species of facultative anaerobes, such as *Pseudomonas aeruginosa* or *Escherichia coli*, can utilize it for anaerobic respiration and growth (Scales et al. 2016; Weinberg et al. 2020). These can disrupt positive processes mentioned earlier (Jin et al. 2019). Moreover, certain lung microbiome signatures may be associated with the risk of recurrence after resection of early stage non-small cell lung cancer (Patnaik et al. 2021). Furthermore, it has been shown that the composition of the lung tissue microbiota may change depending upon the stage of cancer and the presence of metastases (Yu et al. 2016). Similar changes occur in colorectal cancer and could serve as diagnostic or prognostic markers (Villeger et al. 2018). Metagenomic analysis of the microbiome (Yu et al. 2017), presence of certain bacterial species (Villeger et al. 2018; Wong et al. 2017), or analysis of microbiome-related metabolites in feces has been suggested as potential non-invasive methods for early detection or monitoring of colorectal cancer (Villeger et al. 2018).

### Cancer vaccines

Every tumor is characterized by its own unique array of mutations, resulting in a distinctive antigen profile (Sahin and Tureci 2018). Therefore, therapeutic cancer vaccines, administered after the onset of the illness, contrary to most conventional vaccines (Wen et al. 2019), could offer a personalized and targeted method of promoting anti-tumor immune response (Sahin and Tureci 2018). Various vaccine technologies, such as peptides, nucleic acids, recombinant virus, or whole-cell vaccines, are being researched (Thomas and Prendergast 2016). Moreover, they can also contain tumor neoantigens that are already being presented on the surface of in vitro grown dendritic cells (Morse et al. 2021). However, many challenges await on the road to success of cancer vaccines. Only a small fraction of cancer neoantigens is sufficiently immunogenic, a limitation that has to be assessed during cancer vaccine development. Next-generation sequencing enables comprehensive mapping of all mutations in a cancer, which then can be processed by means of bioinformatics, making it possible to predict their immunogenicity (Sahin and Tureci 2018). In vivo immunogenicity is additionally decreased by tumor immunosuppression. Multi-adjuvanted vaccines are a promising method of countering this effect, enabling simultaneous stimulation of immunity and prevention of inhibition (Bowen et al. 2018). This problem can also be addressed by administering a vaccine combining mRNA antigen and immune checkpoint blocking siRNA, delivered directly to immune cells by lipid-coated nanoparticles. It can result in a significant enhancement of immune cell response and profound inhibitory effect on tumor growth and metastasis (Wang et al. 2018). Another challenge is vaccine delivery to the site of cancer. Various nanoparticles, including liposomes, viruses, or polymeric nanoparticles, have been suggested as a feasible solution of this problem (Wen et al. 2019). Currently, there are two cancer vaccines approved for use. Sipuleucel-T, containing autologous peripheral blood mononuclear cells, is a treatment for hormone-refractory metastatic prostate cancer. Bacillus Calmette–Guérin (BCG), containing *Mycobacterium bovis *and commonly used as tuberculosis vaccine, has been approved for treatment of early stage bladder cancer when applied intravesically, which elicits a strong immune response. Many more vaccines are currently undergoing clinical trials (Morse et al. 2021). Other methods, discussed in the following paragraphs, enable overcoming the need for identifying tumor antigens by damaging tumor cells in vivo, which results in in situ vaccination (Locy et al. 2018).

### Oncolytic viruses

Oncolytic viruses (OVs) are attenuated, mutated or benign viruses that target cancer cells, without affecting healthy cells. They are characterized by dual mechanism of action. First, OVs electively replicate in tumor cells, resulting in their destruction, which releases tumor antigens into microenvironment. Second, they simultaneously trigger an inflammatory reaction through the release of damage-associated molecular patterns (DAMPs), which promotes anti-tumor immune response (Marelli et al. 2018). Moreover, it has been shown that this therapy is able to create long-lasting immunological memory, thereby preventing relapse and metastatic spread (Guo et al. 2017). The response to OV therapy can be further increased by engineering them to contain genes coding various proteins stimulating the immune system, such as colony-stimulating factors, interleukins, or chemokines (Marelli et al. 2018). Additionally, OVs targeting the tumor stroma are also being devised, which can inhibit tumor angiogenesis or decrease the density of the stroma, resulting in easier migration of OV, anti-tumor drug, and lymphocyte, towards the tumor (Everts et al. 2020).

One of the biggest challenges for OV treatment is the anti-viral immune response, which, by eliminating the OVs, decreases the anti-tumor response they stimulate. One suggested solution to this problem is sequential treatment with two kinds of OVs. This way, the immune system develops a specific response to the first virus, while enabling the second to exploit its anti-tumor function (Tysome et al. 2012). Another possibility is “Trojan horse” therapy, where immune cells infected with the virus ex vivo deliver it directly to the tumor site, at the same time shielding it from the anti-viral immune response (Marelli et al. 2018).

It has been shown that OVs can improve T-cell infiltration (Ribas et al. 2017) and significantly improve the effectiveness of traditional therapies (Li et al. 2020). They can also be usefully integrated into other tumor immunotherapies (Bommareddy et al. 2018) and even induce therapeutic response in cancer refractory to ICIs (Bommareddy et al. 2019).

### Radiotherapy

Radiotherapy (RT) also greatly influences anti-cancer immune response. It can kill cancer cells, resulting in in situ vaccination and release of pro-inflammatory mediators, increasing tumor-infiltrating immune cells. This makes combining RT with ICIs a promising therapeutic approach (McLaughlin et al. 2020). Moreover, RT might present systemic effects on the immune system, even eliciting immune-mediated systemic tumor regression (Rodriguez-Ruiz et al. 2018). Research suggests that RT may also increase the response to ICIs (Koller et al. 2017). It has been suggested that this interaction is reciprocal, and T-cell activation by immunotherapy may sensitize tumors to radiation treatment by reducing hypoxia and normalizing tumor vasculature (Wang et al. 2019a).

However, RT can also have immunosuppressive effect. Radiation-induced damage to endothelial cells inhibits the infiltration of CD8 + T lymphocytes, activating immunosuppressive pathways, and increases tumor hypoxia, which activates the formation of new blood vessels, induces radioresistance, and limits drug delivery. Between the stimulation of the immune response and the immunosuppression induced by RT is a very delicate balance. Identifying optimal doses of radiation is challenging, since low doses may not damage the entire tumor and high doses can trigger unwanted changes in the TME. Combining RT with ICIs could offer a way to partially overcome these limitations (Wang et al. 2019a; Jarosz-Biej et al. 2019).

### Chimeric antigen receptor T cells

Chimeric antigen receptor (CAR) T-cell therapy represents a major advancement in personalized cancer treatment that has the potential to revolutionize the treatment of lymphomas and possibly other cancers (Neelapu 2019). They are patient’s own T cells, which are genetically engineered to express a synthetic receptor, which enables them to bind tumor antigens in a manner not restricted by major histocompatibility complex (MHC) expression, similar to antibodies. Additional intracellular domains added to the CAR can further strengthen the activation signal. Meaningful tumor regression depends upon CAR T cells’ proliferation and persistence in vivo. However, predictive indicators associated with the success of this therapy are still largely unknown (Feins et al. 2019). Ex vivo assessment of polyfunctionality of CAR T cells, defined as production of multiple cytokines by a single cell, has been suggested to be associated with positive outcomes (Rossi et al. 2018). Upregulation of pathways responsible for exhaustion and apoptosis of T cells, such as PD-L1/PD-1 pathway, has been identified as a possible cause of limited response to CAR T therapy in some tumors. The addition of PD-1 blockade is a promising strategy for overcoming his limitation and enhancing the anti-tumor efficacy of CAR T cells (Wang et al. 2019b). Similar effect can be achieved by PD-1 gene disruption in CAR T cells through genome editing. This novel strategy can significantly improve the anti-tumor activity of this therapy without increasing its toxicity (McGowan et al. 2020).

There are currently two approved CAR T-cell treatments, both targeting the CD19 protein on the surface of B cells, and many more clinical trials are being commenced. However, toxicity is still a barrier to their widespread use (Brudno and Kochenderfer 2019).

### Non-steroidal anti-inflammatory drugs (NSAIDs)

Cyclooxygenase 2 (COX-2)/PGE2 signaling pathway has a strong influence on all hallmarks of cancer (Greenhough et al. 2009). It also plays a role in the inhibition of anti-tumor immune (Nakamura and Smyth 2017). Considering that drugs targeting COX-2 and COX-1, NSAIDs, can be used in cancer pain management (Chapman et al. 2020), it is worth to consider the influence they have on cancer.

Through numerous mechanisms, both COX-dependent and COX-independent, NSAIDs can increase autophagy and apoptosis (Fu et al. 2020b), and decrease inflammation, oxidative stress, angiogenesis, cell migration, and invasion (Vallee et al. 2019). Inhibition of COX-1, through downregulation of thromboxane 2 (TXA2), can inhibit aggregation of platelets on tumor cells, endothelial activation, tumor cell adhesion to the endothelium, and recruitment of TAMs, therefore suppressing the formation of a pre-metastatic niche (Lucotti et al. 2019). A meta-analysis that included over 200,000 participants with prostate, breast, lung, and colorectal cancer from 16 studies showed the potential of NSAIDs to reduce distant metastasis in different types of cancer (Zhao et al. 2017). It has been shown that NSAID use may be associated with improved outcomes even in patients with advanced cancer (Ng et al. 2015).

NSAIDs can also increase the effectiveness of cancer therapy. It has been found that COX-2 expression correlates with and modulates PD-L1 expression in melanoma cells (Botti et al. 2017). Pre-clinical studies seem to confirm that COX inhibition displays synergistic interactions with anti-PD-1 drugs and, therefore, could be considered as promising adjuvants to immune-based therapies (Zelenay et al. 2015).

Chemotherapeutic agents adversely induce COX-2 activity, and NSAID use may help sensitize cancer cells to chemotherapy (Hashemi Goradel et al. 2019). Furthermore, it has been suggested that NSAIDs may inhibit vascular hyper-permeability and hypoxia caused by tumors, and, as a result, normalize pressure gradients across the tumor vessel wall, potentially improving tumor drug delivery (Gkretsi et al. 2017). Similarly to chemotherapy, radiotherapy also induces COX-2 expression in cancer cells. It has been shown that COX-2 inhibitors can sensitize cancer to radiotherapy (Hashemi Goradel et al. 2019). Administered in a perioperative setting, COX-2 inhibitors can also reduce the risk of surgical related metastasis. Importantly, in this case, a clinical benefit was observed with just a short-term treatment at doses with side effects comparable to placebo (Hashemi Goradel et al. 2019; Shaashua et al. 2017).

There are, however, significant limitations to NSAID use in cancer therapy. Its benefits do not come immediately in most cases. Long-term use of NSAIDs can lead to various adverse effects, including bleeding, kidney injury, and cardiovascular incidents. Also, short-term adverse effects limit the dose that can be used. Therefore, both potential risks and benefits need to be carefully considered before including NSAIDs in cancer therapy. Further research is needed to determine their role in cancer treatment and provide clear guidelines for their use in this setting (Chapman et al. 2020; Fu et al. 2020b; Zhao et al. 2017) (see Table 1).

**Table 1:** Novel therapeutic approaches that reshape anti-tumor immune response

Strategy	Brief description	References
Immune checkpoint inhibitors	In normal conditions, immune checkpoint molecules, such as PD-1 and CTLA-4, serve to avoid autoimmunization, but presented on cancer cells prevent their elimination by immune cells. ICI therapy helps to overcome immunosuppression in the TME and improve immune response to the tumor cells	Buchbinder and Desai (2016), Darvin et al. (2018), Postow et al. (2018), Rotte (2019)
Microbiota	Microbiota, through modulating and being modulated by the tumor-associated inflammation, plays a role in diagnosis, prognosis, and various anti-cancer treatments, such as immunotherapy or chemotherapy. It also directly impacts cancer progression and dissemination	Thaiss et al. (2016), Roy and Trinchieri (2017), Villeger et al. (2018), Gopalakrishnan et al. (2018), Sivan et al. (2015), Malczewski et al. (2020), Shang et al. (2020), Scales et al. (2016), Weinberg et al. (2020), Jin et al. (2019), Patnaik et al. (2021), Yu et al. (2016, 2017), Wong et al. (2017), Kim et al. (2019)
Cancer vaccines	As a personalized and targeted approach to enhance anti-tumor immune response, cancer vaccines are administered after the onset of the illness. Despite all the challenges, such as decreased immunogenicity and difficulties in delivery to the tumor site, they constitute a promising therapeutic strategy	Sahin and Tureci (2018), Wen et al. (2019), Thomas and Prendergast (2016), Morse et al. (2021), Bowen et al. (2018), Wang et al. (2018)
Oncolytic viruses	Oncolytic viruses target tumor, but do not attack healthy cells. They replicate in the cancer cells, leading to their damage and activate inflammatory anti-tumor response, which can prevent cancer progression and also contribute to the effectiveness of other treatment strategies	Marelli et al. (2018), Guo et al. (2017), Everts et al. (2020), Tysome et al. (2012), Ribas et al. (2017), Li et al. (2020), Bommareddy et al. (2018, 2019)
Radiotherapy	Radiotherapy has a great influence on the anti-cancer immune response, but the balance between stimulation of the immune activity and immunosuppression is very delicate. Radiation can kill cancer cells and aid in tumor regression, but in other circumstances, it promotes unwanted changes in the TME	McLaughlin et al. (2020), Rodriguez-Ruiz et al. (2018), Koller et al. (2017), Wang et al. (2019a), Jarosz-Biej et al. (2019)
Chimeric antigen receptor T cells	CAR T cells are genetically altered patient’s own T cells, which are able to bind tumor antigens in an MHC-independent manner, which results in activation strengthened by additional intracellular domains. As a personalized tool, they have the potential to revolutionize the treatment of multiple cancer types	Neelapu (2019), Feins et al. (2019), Rossi et al. (2018), Wang et al. (2019b), McGowan et al. (2020), Brudno and Kochenderfer (2019)
Non-steriodal anti-inflammatory drugs	Through numerous mechanisms, NSAIDs decrease inflammation, oxidative stress, cell migration, and invasion, and promote DNA repair, cell cycle arrest, cell differentiation, and apoptosis, hence their anti-tumoral effect. It has been shown that NSAID use may be associated with an improved outcome even in advanced cancer and an increase in effectiveness of other therapies	Vallee et al. (2019), Lucotti et al. (2019), Ng et al. (2015), Botti et al. (2017), Zelenay et al. (2015), Robledo-Cadena et al. (2020)

## Conclusions

Tumor-associated inflammation plays a crucial role in cancer progression and treatment. It is a remarkably complex process, involving numerous cell populations, each of them playing a distinct role, and influencing all aspects of tumor biology, including growth, progression, metastasis, and response to treatment. Although the naturally occurring inflammation at the tumor site often promotes cancer progression, it can be remodeled to take on anti-tumor properties. This can enable the development of new cancer medicines and improvement of the effectiveness of existing therapies, at the same time decreasing the intensity of their adverse effects. Therefore, further research in this field is crucial to better understand the intricacies of cancer microenvironment and immune response, and to optimize cancer treatment.
